# Functionalized single-walled carbon nanotubes/polypyrrole composites for amperometric glucose biosensors

**DOI:** 10.1186/1556-276X-8-316

**Published:** 2013-07-09

**Authors:** Matei Raicopol, Alina Prună, Celina Damian, Luisa Pilan

**Affiliations:** 1 Faculty of Applied Chemistry and Materials Science, University Politehnica of Bucharest, 132 Calea Griviţei, 010737 Bucharest, Romania; 2 Faculty of Physics, University of Bucharest, 405 Atomistilor, Magurele, Romania

**Keywords:** Functionalization, Carbon nanotubes, Conductive polymers, Nanocomposite, Amperometric biosensor, Sensitivity, Selectivity

## Abstract

This article reports an amperometric glucose biosensor based on a new type of nanocomposite of polypyrrole (PPY) with *p*-phenyl sulfonate-functionalized single-walled carbon nanotubes (SWCNTs-PhSO_3_^−^). An environmentally friendly functionalization procedure of the SWCNTs in the presence of substituted aniline and an oxidative species was adopted. The nanocomposite-modified electrode exhibited excellent electrocatalytic activities towards the reduction or oxidation of H_2_O_2_. This feature allowed us to use it as bioplatform on which glucose oxidase (GOx) was immobilized by entrapment in an electropolymerized PPY/SWCNTs-PhSO_3_^−^ film for the construction of the glucose biosensor. The amperometric detection of glucose was assayed by applying a constant electrode potential value necessary to oxidize or reduce the enzymatically produced H_2_O_2_ with minimal interference from the possible coexisting electroactive compounds. With the introduction of a thin film of Prussian blue (PB) at the substrate electrode surface, the PPY/GOx/SWCNTs-PhSO_3_^−^/PB system shows synergy between the PB and functionalized SWCNTs which amplifies greatly the electrode sensitivity when operated at low potentials. The biosensor showed good analytical performances in terms of low detection (0.01 mM), high sensitivity (approximately 6 μA mM^−1^ cm^−2^), and wide linear range (0.02 to 6 mM). In addition, the effects of applied potential, the electroactive interference, and the stability of the biosensor were discussed. The facile procedure of immobilizing GOx used in the present work can promote the development of other oxidase-based biosensors which could have a practical application in clinical, food, and environmental analysis.

## Background

Research and development in electrochemical biosensors have gained increasing importance as analytical tools in the last years, since electrochemical biosensors have advantageous properties such as the simplicity of use, potential miniaturization, and low cost, in comparison with well-established, lab-based methods. However, a number of problems are still present, preventing the total success in the sensor market, so nanocomposite materials may play an important role for improving their properties [1].

Conducting polymers (CPs) are especially amenable to the development of electrochemical biosensors by providing biomolecule immobilization and rapid electron transfer. The combination of known CP substrates with carbon nanotubes (CNTs) may generate composites with new and interesting properties, providing higher sensitivity and stability of the immobilized molecules, thus constituting the basis for new and improved analytical devices for biomedical and other applications [2]. The enhanced response can be attributed to several factors such as the improved electron transfer within the polymeric matrix from the presence of CNTs, the direct electron transfer from the active site of the enzymes to the electrode through the CNTs bridging them, and the enhanced accessibility of the enzyme catalytic sites for the substrate due to highly open reticular morphology of the nanocomposite film. Surface functionalization of CNTs can greatly enhance their utility in the formation of composites by aiding in dispersability and ensuring efficient interactions between the SWCNTs and the host materials [3]. In this regard, the development of simple and cost-effective chemical procedures for covalent functionalization of CNTs is a matter of increasing importance [4]. In our research an environmentally friendly functionalization procedure of the SWCNTs was adopted. The reaction was performed ‘on water’ in the presence of a substituted aniline and an oxidative species similar to that described by Price and Tour [5] with obtainment of *p*-phenyl sulfonate-functionalized SWCNTs (SWCNTs-PhSO_3_^−^). Running reactions on water can reduce harmful waste and reaction times while increasing yields and reaction rates [5].

Among the various conducting polymers, films of PPY and derivatives have good conductivity, selectivity, stability, and efficient polymerization at neutral pH [6]. Enzymes and, in particular, oxidases, have been preferentially chosen for the entrapment in PPY matrices, but other biomolecules are also potential targets. In general, glucose oxidase (GOx) is selected as a model enzyme due to its low cost, stability, and practical utility. The oxidases act by oxidizing the substrate and then returning to their original active state by transferring electrons to molecular oxygen, so the final products of these enzymes are the oxidized form of the substrate and, as a side product, hydrogen peroxide (H_2_O_2_). Both the measurement of oxygen consumption and H_2_O_2_ production can provide information about the concentration of the enzyme substrate (glucose). Methods based on the measurement of H_2_O_2_ have been greatly preferred in the recent years to those based on the reduction of oxygen. However, a great drawback in this approach is represented by the high overpotential needed for H_2_O_2_ oxidation (greater than +0.6 V vs. Ag/AgCl reference electrode). At this relatively high potential, there may be interferences from other oxidable species such as ascorbic acid, uric acid, and acetaminophen. One of the most common ways to overcome this problem has been the use of another enzyme, namely, horseradish peroxidase (HRP) which catalyzes the reduction of H_2_O_2_ and allows the direct electron transfer between its active site and the electrode surface [7]. This approach, although exhibiting good sensitivity and accuracy, suffers from some important shortcomings such as high cost, low stability, and the limited binding of HRP to solid surfaces. For this reason, the electrochemical inorganic mediators [8], able to catalyze the oxidation or reduction of hydrogen peroxide, have been preferred to HRP and have been used for the assembling of oxidase-based biosensors. This results in a decrease of the applied potential and the consequent avoidance of many electrochemical interferences. In this perspective, Prussian blue (PB), which has high electrocatalytic activity, stability, and selectivity for H_2_O_2_ electroreduction, has been extensively studied and used for H_2_O_2_ detection [9].

Incorporating a thin PB film into the PPY/GOx/SWCNTs-PhSO_3_^−^ nanocomposite, the obtained hybrid shows synergistic augmentation of the response current for glucose detection. The effects of applied potential on the current response of the composite-modified electrode toward glucose, the electroactive interference, and the stability were optimized to obtain the maximal sensitivity. The resulting biosensor exhibits high sensitivity, long-term stability, and freedom of interference from other co-existing electroactive species.

## Methods

### Chemicals and instrumentation

Single-walled carbon nanotubes (>90% C, >77% C as SWCNTs) were obtained from Aldrich (Sigma-Aldrich Corporation, St. Louis, MO, USA). Glucose oxidase (type X-S from *Aspergillus niger*, 250,000 μg^−1^) was purchased from Sigma. Pyrrole (98%, Aldrich), D-(+)-glucose (≥99.5%), ascorbic acid, uric acid, and acetaminophen were used as received (Sigma). All other chemicals were of analytical grade. Electrochemical experiments were performed using a 128N Autolab potentiostat and a conventional three-electrode system with a platinum-modified electrode (disk-shaped with diameter of 2 mm; Metrohm Autolab B.V., Utrecht, the Netherlands) as the working electrode, a platinum wire as the counter electrode, and Hg/Hg_2_Cl_2_ (3 M KCl) as reference electrode (purchased from Metrohm). Unless otherwise stated, all experiments were carried out at room temperature in pH 7.4 phosphate buffer solution (0.1 M phosphate). Amperometric determination of glucose was carried out at different applied potentials under magnetic stirring.

### Single-walled carbon nanotubes functionalization

**Figure 1:**
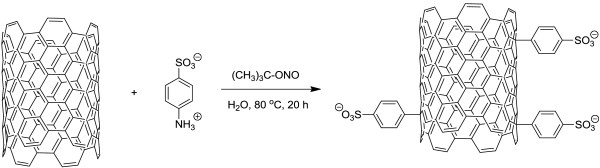
Functionalization of SWCNTs.

For the functionalization of SWCNTs, we have adopted a procedure similar to that described by Price and Tour [5] with minor modifications as presented in Figure 1. Twenty-five milligrams of SWCNTs was dispersed in 50 mL deionized water using a high-shear homogenizer at 10,000 rpm for 30 min. The resulting suspension was transferred to a round-bottom flask fitted with a magnetic stirrer and condenser and 1.44 g sulfanilic acid (Fluka Chemical Corporation, St. Louis, Milwaukee, WI, USA) followed by addition of 0.52 mL *tert*-butyl nitrite (Aldrich). The reaction mixture was stirred at room temperature for 30 min then the temperature was increased to 80°C and maintained for 20 h. After that acetone was added, and the CNTs were filtered on Teflon membrane filter (0.2-mm pores) and washed with water and then acetone. The CNTs were then dispersed in 5 mL of *N*,*N*-dimethylformamide using an ultrasonic bath and precipitated again with acetone, filtered, and washed with acetone. Finally, the CNTs were treated with diluted NaOH solution (30 min in the ultrasonic bath), filtered, washed with water followed by acetone, and dried.

### Preparation of the modified electrode

Firstly, a PB film was electropolymerized at the Pt electrode surface in an unstirred fresh 2 mM K_3_Fe(CN)_6_ + 2 mM FeCl_3_. 6H_2_O in 0.1 M KCl + 1 mM HCl aqueous solution by cyclic voltammetry in the potential range of −0.2 to 1.0 V at a scan rate of 0.1 V s^−1^.

Different amounts of the functionalized nanotubes (usually 1 mg/mL) were dispersed in bidistilled water by sonication for 1 h. The selected amount of GOx (1 mg/mL) was then added to the CNTs solution. Afterwards, pyrrole was added (at a concentration of 0.5 M) to the GOx and SWCNTs-PhSO_3_^−^ mixture, and the electropolymerization was performed at current densities of 0.1, 0.2, or 0.5 mA cm^−2^ for different times. The electropolymerization was carried out at pH 7.4. After the electropolymerization, the composite film (PPY/GOx/SWCNTs-PhSO_3_^−^/PB) was subjected to overoxidation by cycling the potential from −0.2 to 1 V for 50 cycles at 0.1 V s^−1^ in a phosphate buffer solution at pH 7.4. For comparison, PPY/GOx/SWCNTs-PhSO_3_^−^, PPY/GOx/PB, and PPY/GOx films have been also obtained.

## Results and discussion

**Figure 2:**
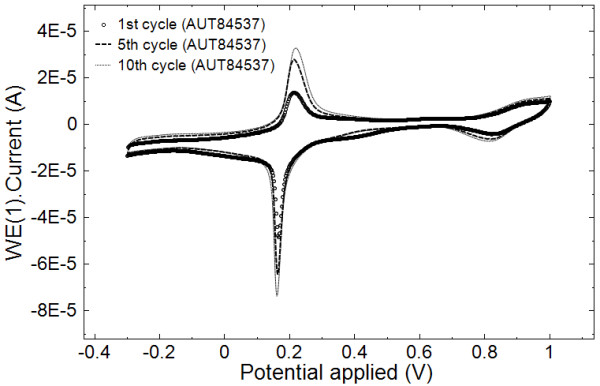
Cyclic voltammograms of Prussian blue film electrosynthesis at Pt electrode.

PB-modified electrodes have been synthesized by the simple and versatile electrochemical method proposed by Itaya et al. [10] based on the reduction of a ferric-ferricyanide solution as described in the ‘Methods’ section. The procedure can be adopted with different electrode materials (platinum, gold, and glassy carbon), and a high stability of the layer deposited through successive cycling was demonstrated [10]. The typical cyclic voltammogram recorded during PB film electrosynthesis, as described in the Methods section, is shown in Figure 2. Two sets of peaks can be observed in cyclic voltammetry recordings for PB/Pt-modified electrodes synthesis which correspond to the reduction and oxidation of PB to Prussian white (*E*_1/2_ = 0.2 V) and to Berlin green (*E*_1/2_ = 0.9 V), respectively.

**Figure 3:**
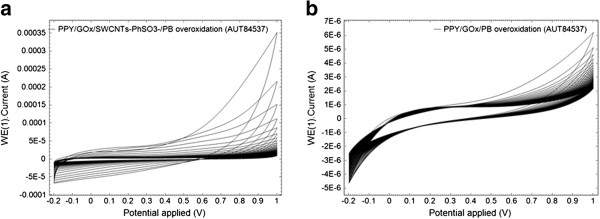
**Cyclic voltammograms corresponding to overoxidation.** PPY/GOx/SWCNTs-PhSO_3_^−^/PB **(a)** and PPY/GOx/PB **(b)** films in a 0.1 M phosphate buffer solution (pH 7.4), for a scan rate of 0.05 V s^−1^.

**Figure 4:**
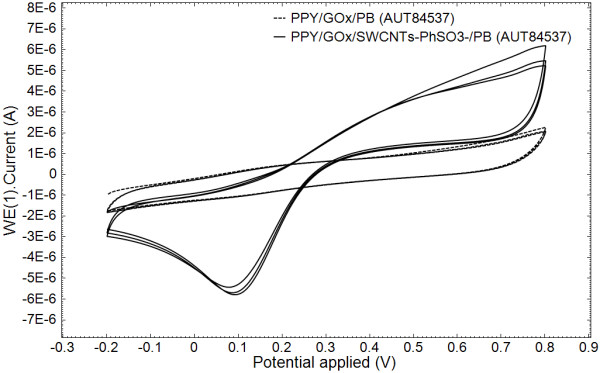
**Cyclic voltammograms at the PPY/GOx/SWCNTs-PhSO**_**3**_^**−**^**/PB/Pt and PPY/GOx/PB/Pt electrodes.** Cyclic voltammograms at the PPY/GOx/SWCNTs-PhSO_3_^−^/PB/Pt and PPY/GOx/PB/Pt electrodes (previously subjected to 50 overoxidation cycles) in a 0.1-M phosphate buffer solution (pH 7.4), for a scan rate of 0.05 V s^−1^.

Then the pyrrole electropolymerization was carried out galvanostatically at PB/Pt electrode surface. The electropolymerization was performed in 0.1 M phosphate buffer solution at a pH of 7.4, above the isoelectric point of the glucose oxidase, in order to provide an overall negative charge so that the glucose oxidase can electrostatically attach to the PPY backbone. The overoxidation of enzyme-doped PPY electrodes leads to a loss of the PPY electroactivity and to an enhanced sensitivity and selectivity to glucose. In Figure 3, the overoxidation cyclic voltammograms for the composite (Figure 3a) and pure polymeric (Figure 3b) PPY films show evidence of the clear changes of the film voltammetric characteristics upon incorporation of nanotubes as a fixed dopant. The same conclusion results from the analysis of Figure 4 where the cyclic voltammetry investigation of the PPY/GOx/PB film and the PPY/GOx/SWCNTs-PhSO_3_^−^/PB composite film (obtained in the same conditions and after overoxidation) is shown. It can be observed that the SWCNTs-PhSO_3_^−^ counter ion has a marked effect on the properties of the resulting PPY/GOx/SWCNTs-PhSO_3_^−^/PB film, such as improved capacitance. The background current of PPY/GOx/SWCNTs-PhSO_3_^−^/PB is larger than that of PPY/GOx/PB, which indicates that the nanocomposite-modified electrode has larger effective surface area.

### Raman spectroscopy characterization

**Figure 5:**
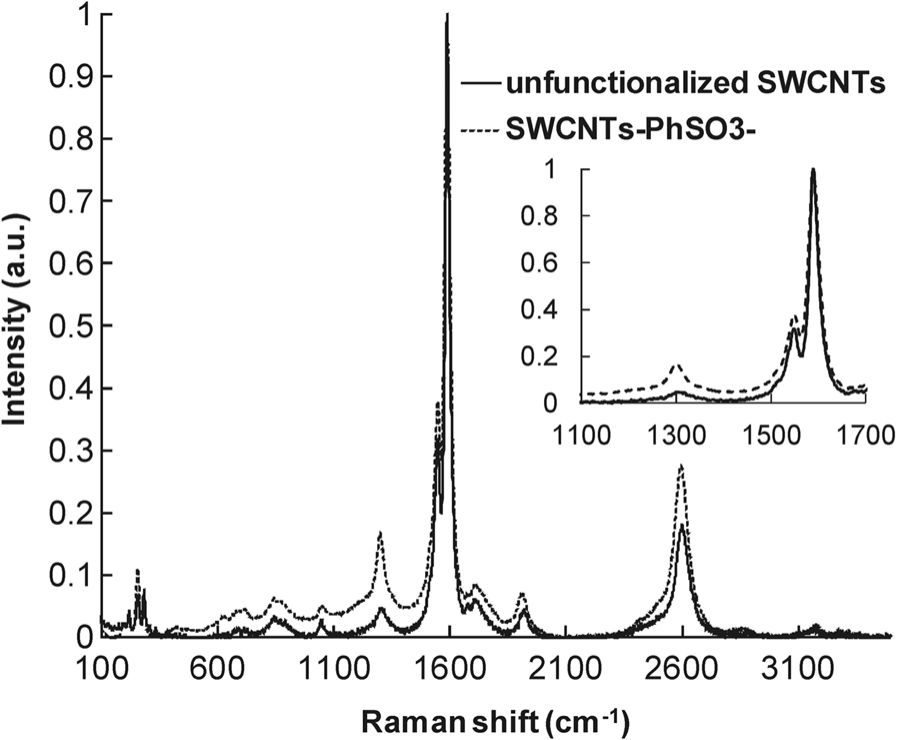
Raman spectra of as received and functionalized SWCNTs.

The functionalized SWCNTs were characterized using Raman spectroscopy, a method commonly utilized in SWCNTs analysis. The spectra of the studied SWCNTs samples for an excitation wavelength of 633 nm with a magnification of the ‘G’ and ‘D’ bands frequency range are shown in Figure 5. The Raman spectra of the starting material (unfunctionalized SWCNTs) show a disorder mode (diamondoid or D band) with a very low intensity at 1,300 cm^−1^. The spectra of SWCNTs-PhSO_3_^−^ material show an increased intensity in the disorder mode, indicating functionalization of the SWCNTs. The increase in the D band is attributed to the *sp*^3^ carbons present in the SWCNTs after functionalization. The relative degrees of functionalization can be evaluated by dividing the intensity of the disorder mode by the intensity of the tangential mode (graphitic or G band) at 1,591 cm^−1^.

**Figure 6:**
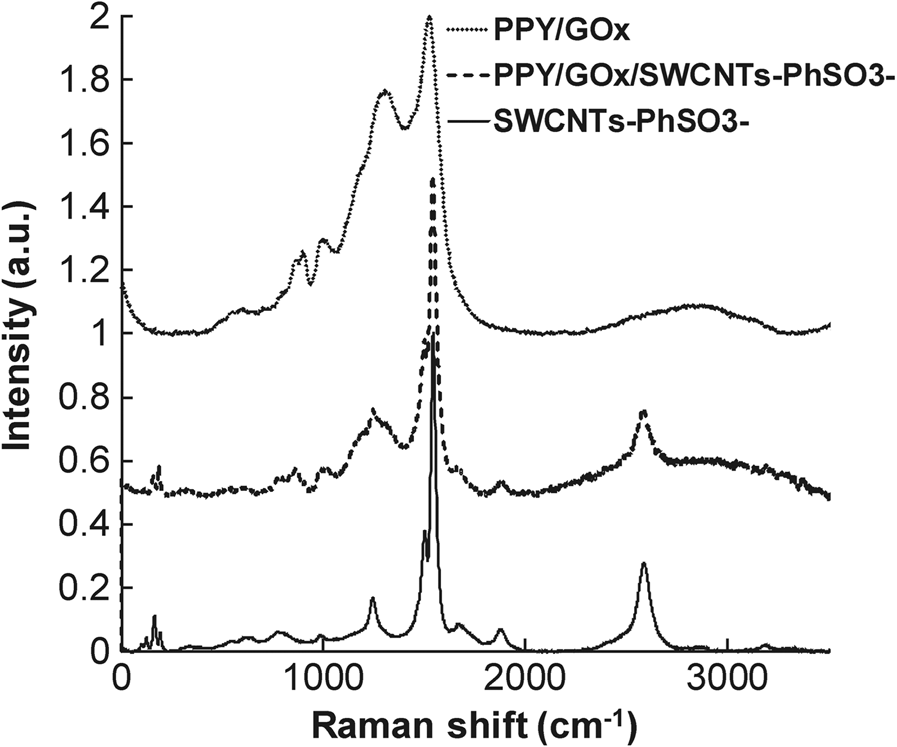
Raman spectra of the electrochemically deposited polymeric films in comparison with the functionalized SWCNTs.

Figure 6 presents the Raman spectra of PPY/GOx and PPY/GOx/SWCNTs-PhSO_3_^−^ composite (obtained galvanostatically at 0.1 mA cm^−2^ for 20 min). For comparison, the spectrum of SWCNTs-PhSO_3_^−^ is also shown in this figure, which contains the two strong peaks at 1,300 and 1,591 cm^−1^. For PPY and PPY/GOx/SWCNTs-PhSO_3_^−^ composites, the peaks at 933 and 1,080 cm^−1^ have been associated with the quinonoid bipolaronic structure and those at 980 and 1,055 cm^−1^ with the quinonoid polaronic structure, revealing the presence of the doped PPY structures [11]. The peak at 1,320 cm^−1^ designates antisymmetrical C-H in-plane bending, and the strong peak at 1,588 cm^−1^ represents the backbone stretching mode of C=C bonds. Obviously, the Raman spectra of the nanocomposites present the characteristics both those of PPY/GOx and functionalized SWCNTs, indicating that the SWCNTs serve as the core in the formation of a tubular shell of PPY/GOx/SWCNTs-PhSO_3_^−^ composites.

The Raman spectra of electrochemically deposited PPY/GOx/SWCNTs-PhSO_3_^−^ composite are strongly dependent on different parameters such as electrodeposition time or density current. In some samples of PPY/GOx/SWCNTs-PhSO_3_^−^ composite (higher current densities used for electrodeposition), the Raman spectra are quite modified from the CNT spectra: the lines corresponding to the breathing mode disappear. This maybe because the PPY was too thick in the used samples. Further work is in progress in order to characterize the samples and correlate their properties with the electrochemical parameters used during synthesis.

### SEM characterization

**Figure 7:**
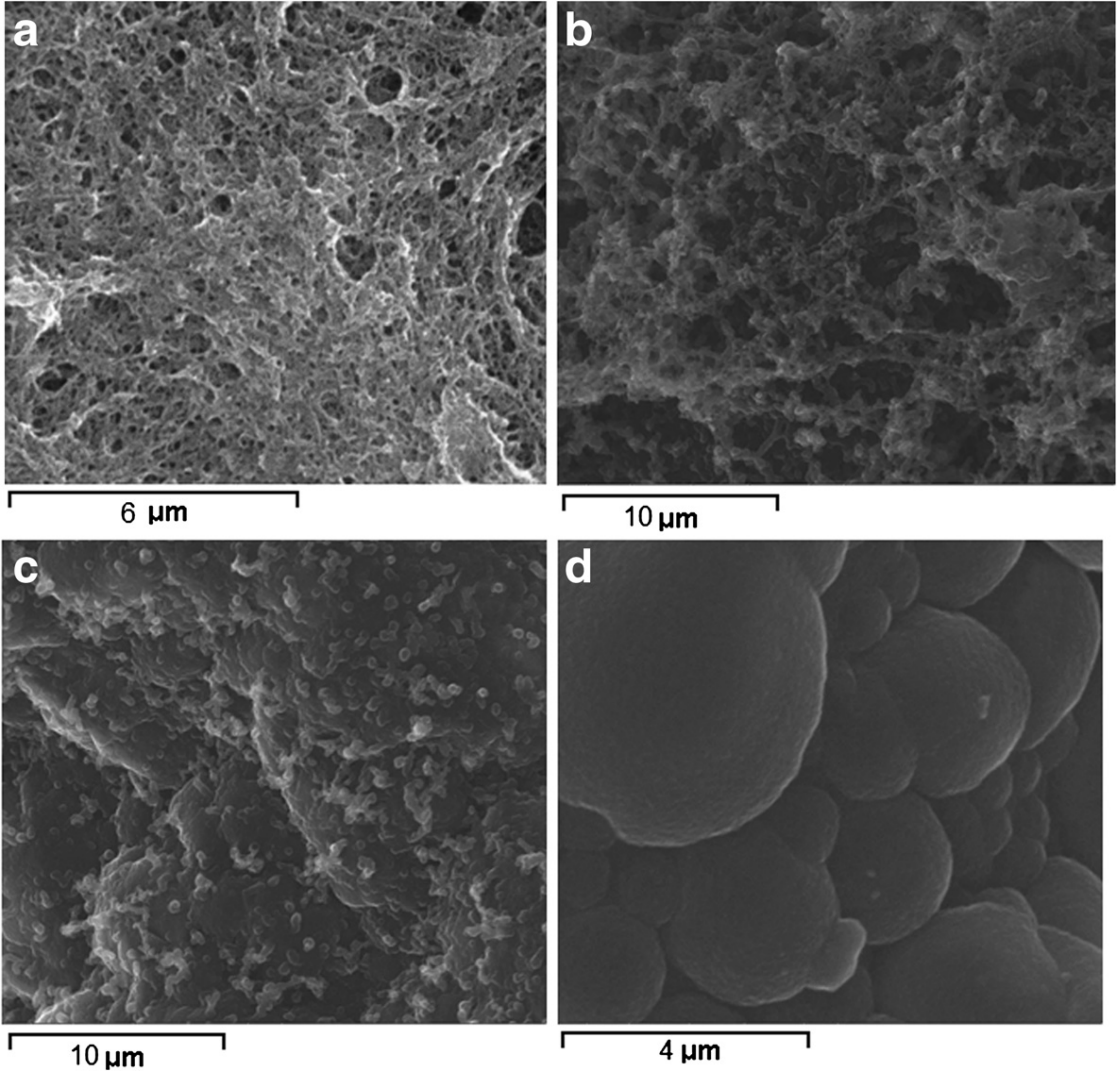
**SEM images.** Functionalized SWCNTs **(a)**, PPY/GOx/SWCNTs-PhSO_3_^−^ composite films obtained galvanostatically at 0.1 mA cm^−2^**(b)** and 0.5 mA cm^−2^**(c)**, and pure polymeric PPY **(d)**.

The surface morphology of the films differs remarkably between the PPY/GOx/SWCNTs-PhSO_3_^−^ and pure polymeric PPY films (Figure 7). Scanning electron microscopy (SEM) image of PPY/GOx/SWCNTs-PhSO_3_^−^ film reveals a very fibrous three-dimensional reticular structure with interlocking pores unlike the PPY typical cauliflower morphology. The diameter of the PPY/GOx/SWCNTs-PhSO_3_^−^ fibrils is significantly larger than that of the SWCNTs-PhSO_3_^−^ and this indicates a good interaction between the functionalized SWCNTs and pyrrole monomer. The functionalized SWCNTs acted as a dopant and also provided a large surface area for the polymerization process to take place. It can be stated that a three-dimensional network was formed with the functionalized SWCNTs serving as the backbone. The improved electrochemical properties for the PPY/GOx/SWCNTs-PhSO_3_^−^ film can be also explained by this porous morphology of the composite film that provides enough pathways for the movement of ions and solvent molecules within the film.

### Biosensor performance

**Figure 8:**
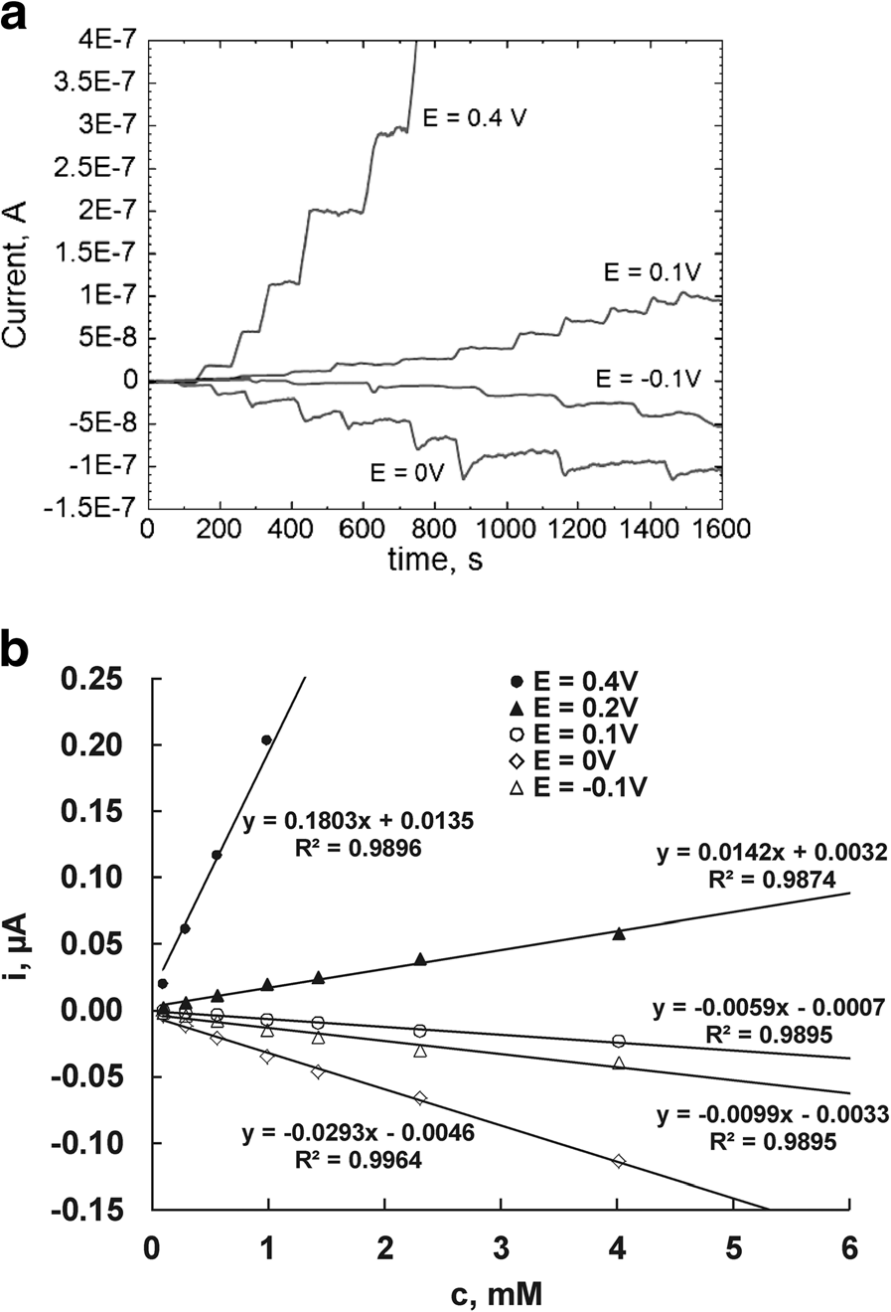
**Current-time recordings for successive additions of glucose in 0.1 M phosphate buffer solution pH 7.4.** Current-time recordings for successive additions of glucose in 0.1 M phosphate buffer solution pH 7.4 at PPY/GOx/SWCNTs-PhSO_3_^−^/PB/Pt-modified electrode measured at different applied potentials **(a)** and the corresponding calibration plots (linear region) for the sensing of glucose using PPY/GOx/SWCNTs-PhSO_3_^−^/PB/Pt nanocomposite-modified electrode **(b)**.

The effect of applied potential on the amperometric response of the PPY/GOx/SWCNTs-PhSO_3_^−^/PB/Pt biosensor was studied. Amperometric measurements were performed in stirred 0.1 M phosphate buffer pH 7.4 solution by injecting different quantities of 10 mM and 0.1 M glucose solution after baseline stabilization at each applied potential. The amperometric responses of the PPY/GOx/SWCNTs-PhSO_3_^−^/PB/Pt electrode related to the glucose concentration over the 0.4 to −0.1 V vs. Hg/Hg_2_Cl_2_(3 M KCl) range of applied potentials are illustrated in Figure 8a. The optimal detection potential in terms of both sensitivity and selectivity was 0 V. The typical calibration curve of the biosensor based on PPY/GOx/SWCNTs-PhSO_3_^−^/PB/Pt electrode for glucose is linear with the glucose concentration up to 6 mM (Figure 8b), and then a plateau is reached gradually at higher glucose concentration (not shown). The PPY/GOx/SWCNTs-PhSO_3_^−^/PB/Pt electrode has a detection limit of 0.01 mM, higher than compared with PPY/GOx/SWCNTs-PhSO_3_^−^/Pt biosensor (0.05 mM), and has a larger response current. On the other hand, the linear range is narrower when compared with PPY/GOx/SWCNTs-PhSO_3_^−^/Pt (up to 10 mM), which is similar to that reported for PB-modified biosensors [12].

**Figure 9:**
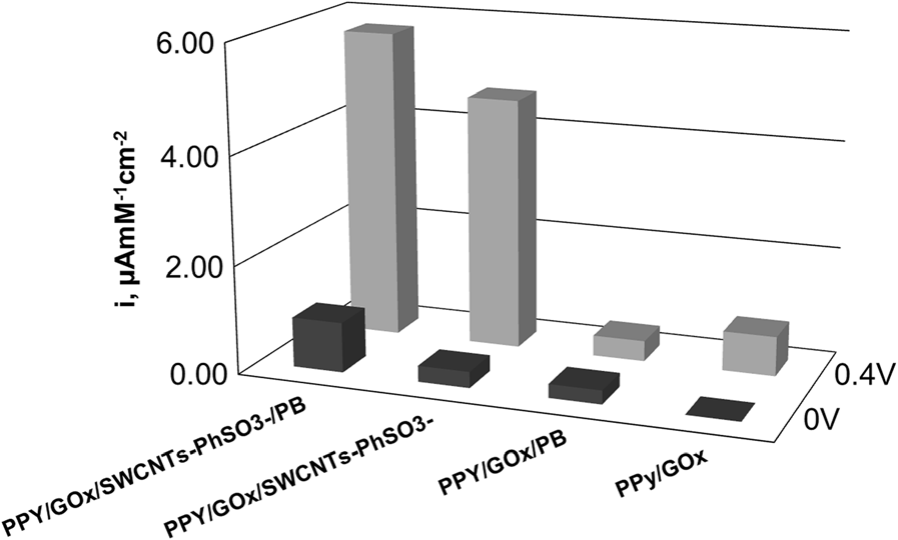
**Comparative sensitivities for PPY/GOx/SWCNTs-PhSO**_**3**_^**−**^**/PB/Pt, PPY/GOx/SWCNTs-PhSO**_**3**_^**−**^**/Pt, PPY/GOx/PB/Pt and PPY/GOx/Pt for 0 and 0.4 V operation potentials.**

The concluded analytical data (sensitivities) for the studied biosensors obtained from the calibration curves are presented in Figure 9. The PPY/GOx/SWCNTs-PhSO_3_^−^/PB/Pt biosensor displayed superior sensitivities to those documented in literature for PPY/GOx/CNTs composites: 0.44 μA mM^−1^[12], 2.33 nA mM^−1^[13], 0.28 μA mM^−1^[14,15], 80 nA mM^−1^ cm^−2^[16], and 0.016 μA mM^−1^ cm^−2^[17].

The low operation potential afforded by the PPY/GOx/SWCNTs-PhSO_3_^−^/PB/Pt biosensor greatly minimizes the contributions from easily oxidizable compounds which commonly interfere with the biosensing of glucose. The effects of ascorbic acid, acetaminophen, and uric acid upon the response of the glucose biosensor were evaluated at the operation potential of 0 V. It was found that the addition of 0.2 mM ascorbic acid, 0.1 mM acetaminophen, and 0.5 mM uric acid to 2 mM of glucose solution did not cause any impact on the response of the biosensor (Table 1).

**Table 1:** **Influence of electroactive interferents on glucose response at PPY/GOx/SWCNTs-PhSO**_**3**_^**−**^**/PB/Pt electrode**

**Interferent**	**Concentration (physiological normal, mM)**	***i***_**Glu + interf**_**/*i***_**Glu**_^**a **^**at *E *= 0 V**
Ascorbic acid	0.2	1.07
Acetaminophen	0.1	1.05
Uric acid	0.5	1.03

^a^*i*_Glu_ is the response current to 2 mM glucose; *i*_Glu + interf_ is the response current to 2 mM glucose in presence of interferent at physiological normal concentration. Results are obtained at the operation potential of 0 V.

The storage stability of the biosensor was also studied. The steady-state response current of 2 mM glucose was determined every 2 days. When not in use, the biosensor was stored in 0.1 M phosphate buffer pH 7.4 at 4°C. The results show that the steady-state response current only decreases by 12% after 30 days measurements, which indicates that the enzyme electrode was considerably stable.

## Conclusions

A new type of PPY/GOx/SWCNTs composite has been obtained in a one-step preparation procedure by electropolymerization of pyrrole in the presence of enzyme and functionalized SWCNTs. For SWCNTs-PhSO_3_^−^ synthesis, an environmentally friendly functionalization procedure was adopted. The reaction was performed on water in the presence of sulfanilic acid and *tert*-butyl nitrite. The functionalized SWCNTs were characterized using spectroscopic and microscopic methods.

The studies undertaken in this article demonstrate that the new electrochemically synthesized PPY/GOx/functionalized SWCNTs nanocomposite can be used for the fabrication of electrochemical glucose biosensors with attractive performance. The nanocomposite biosensor exhibits high sensitivity and low detection limits even at an applied potential of 0 V vs. Hg/Hg_2_Cl_2_ (3 M KCl). The performance in glucose determination is better than that of much more biosensor assemblies based on similar components. The glucose biosensor shows good analytical characteristics such as low detection limit (0.01 mM), high sensitivity (approximately 6 μA mM^−1^ cm^−2^), wide linear range (0.02 to 6 mM), and good stability under the optimized experimental conditions. The selectivity of the biosensor is greatly improved due to the lower operation potential afforded by the catalytic ability of the presence of both PB film and SWCNTs. The PPY/GOx/SWCNTs-PhSO_3_^−^/PB hybrid material has a potential to provide operational access to a large group of oxidase enzymes for designing a variety of biosensing devices.

## Competing interests

The authors declare that they have no competing interests.

## Authors' contributions

LP and MR wrote the paper and performed electrochemistry and organic synthesis experiments, respectively. AP and CD performed some additional experiments followed by data analysis and helped during the manuscript preparation. LP and AP incorporated the final corrections into the manuscript. All authors read and approved the final manuscript.
